# Identification of key elements in MRI reporting of intracranial meningiomas based on a nationwide survey of clinical experts in Germany

**DOI:** 10.1038/s41598-024-83737-1

**Published:** 2025-01-07

**Authors:** Torge Huckhagel, Tammam Abboud, Jan Regelsberger, Stefan Rieken, Christian Riedel

**Affiliations:** 1 Department of Diagnostic and Interventional Neuroradiology, University Medical Center Göttingen, Robert-Koch-Straße 40, 37075 Göttingen, Germany; 2 Department of Neurosurgery, University Medical Center Göttingen, Göttingen, Germany; 3 Department of Neurosurgery, Diako Krankenhaus Flensburg, Flensburg, Germany; 4 Department of Radiotherapy and Radiation Oncology, University Medical Center Göttingen, Göttingen, Germany

**Keywords:** Diagnostic imaging, Magnetic resonance imaging, Meningeal neoplasms, Meningioma, Cancer imaging, CNS cancer

## Abstract

While MRI has become the imaging modality of choice for intracranial meningiomas, no radiologic reporting guidance exists to date that relies on a systematic collection of information relevant to the core medical disciplines involved in the management of these patients. To address this issue, a nationwide expert survey was conducted in Germany. A literature-based catalog of potential reporting elements for MRI examinations of meningioma patients was developed interdisciplinarily. Subsequently, all board-certified members of the German Societies of Neuroradiology, Neurosurgery and Radiation Oncology with expertise in managing meningioma patients were invited to vote on the relevance of the suggested items via online survey. A total of 150 experts participated in the study (104 neurosurgeons/radiation oncologists, 46 neuroradiologists). The reporting elements of tumor location, extent, growth pattern, contrast uptake, associated cysts, and impact on adjacent anatomic structures received widespread approval (> 75.0% of all participants). In addition, a vast majority (> 75.0%) supported reference to perifocal edema, signs of mass effect, and hydrocephalus. Postoperative imaging is particularly requested to describe the extent of resection (94.0%) and treatment-related changes (89.3%). Advanced methods (diffusion, perfusion, proton spectroscopy) and meningioma-specific classifications (Nauta, Zee, Sindou) were judged to be less relevant (< 50.0% agreement) to MRI reporting. To serve as a vital clinical communication tool and enable an optimal contribution to the care of meningioma patients, the radiological report should focus on the fundamental information requirements of the neuro-oncology treatment team encompassing primarily tumor location, extent, tissue imaging characteristics, and potential impairment of neighboring anatomical structures.

## Introduction

According to the most recent Central Brain Tumor Registry of the United States (CBTRUS) report, meningiomas are the most commonly encountered histopathology among all primary brain and other central nervous system (CNS) tumors, accounting for approximately 40% of all cases in the United States. Most of these predominantly benign (WHO CNS 1/2) tumors, which are more common in females and have an incidence increasing with age, are found in the cerebral meninges (82%)^1^. The unique role of neuroimaging and the associated responsibility of the (neuro)radiologist regarding meningiomas in contrast to malignant CNS tumors (e.g., glioblastomas) results from the fact that in the case of an incidental and asymptomatic finding, a provisional radiological diagnosis is made, and magnetic resonance imaging (MRI) follow-up is the guideline-based management strategy of choice instead of immediate histological confirmation^2^. Thin-slice MRI is generally recommended by the Response Assessment in Neuro-Oncology Working Group (RANO) as the standard imaging method of choice for radiologic workup of meningiomas because of its superior sensitivity and reproducibility, although computed tomography with bone window setting has additional value in assessing hyperostosis of the adjacent bony structures and intraosseous tumor infiltration, respectively^2,3^. Most authors base their suggestions for MRI examinations of patients with intracranial meningiomas on the established standardized Brain Tumor Imaging Protocol (BTIP) originally developed for gliomas, which requires at least three-dimensional isotropic T1-weighted sequences before and after contrast administration, axial two-dimensional T2 FLAIR and T2-weighted sequences, as well as an axial diffusion sequence^3–5^. On the other hand, alternative strategies avoiding contrast media are being explored due to the need for repeated follow-up scans and the concerns about potential risks of gadolinium-based contrast agents including allergy-like reactions, nephrogenic systemic fibrosis and gadolinium deposition in certain brain regions, although there is currently no proven evidence of adverse health effects from these deposits^6^. This approach is supported by the demonstrated strong correlation between meningioma size in pre-contrast T2-weighted images and post-contrast T1-weighted images^7^. Regarding treatment response, the RANO Working Group has proposed criteria for clinical trials that assess meningioma burden over time based on changes in the products of the maximal cross-sectional enhancing diameters. These criteria are comparable to the percentage-based categories of the updated RANO 2.0 recommendations for gliomas but, in contrast to the latter, exclusively consider the contrast-enhancing tumor tissue^3,8^. While numerous review articles and authoritative references have detailed the MRI characteristics of both common (meningothelial, fibrous, or transitional) and rare subtypes of meningiomas from a neuroradiological perspective^5,9–12^, comprehensive insights into the informational needs of clinicians involved in the therapeutic management of these patients are still lacking. Therefore, the aim of this study was to address this issue through a nationwide survey on content priorities of meningioma MRI reports conducted among neurosurgeons, radiation oncologists, and neuroradiologists in the Federal Republic of Germany, as surgery and irradiation are the current cornerstones of treatment for these tumors^2,13^.

## Methods

### Ethics and study design

The study protocol was assessed by the Institutional Ethics Committee of the University Medical Center Göttingen (registration number 9/2/21), and ethics approval was not required since it does not involve medical research on human subjects and thus does not necessitate formal approval. The research entails a nationwide structured online survey conducted among board-certified specialists in neurosurgery, radiation oncology, and neuroradiology in Germany, who regularly treat or diagnose patients with intracranial meningiomas. Data were gathered prospectively through a cross-sectional poll. Informed consent was obtained from all participating experts. Whenever possible and appropriate, the study design and presentation of results adhere to the recommendations for conducting and reporting survey research as proposed by Kelley and colleagues^14^.

### Course of study

**Fig. 1:**
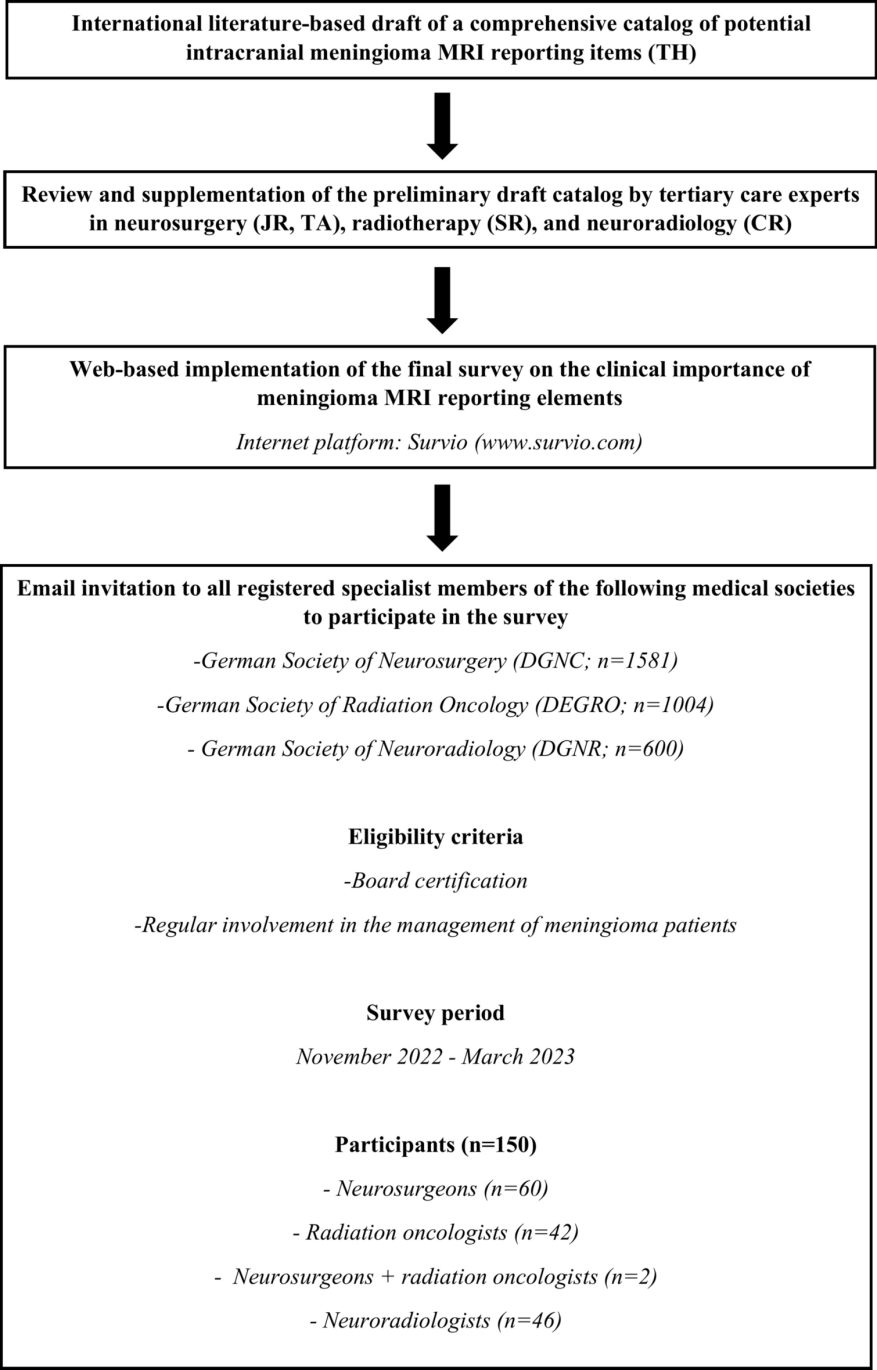
Flowchart describing the study course, study period, and eligibility criteria of the surveyed clinical experts.

Based on an extensive review of both international English-language and German-language neuroradiological, neurosurgical, and radiation oncology literature, the first author (TH), a board-certified neurosurgeon with several years of additional experience in clinical neuroradiology, compiled a comprehensive catalog of potential radiology reporting items for MRI examinations in patients with intracranial meningiomas. Supplement ‘references for meningioma mri reporting items’ provides selected references for all reporting elements included in the catalog. The preliminary draft underwent rigorous assessment for clarity and completeness by all co-authors, who are experienced specialists in neurosurgery (JR, TA), radiotherapy (SR), and radiology with a specialization in neuroradiology (CR). After incorporating their feedback, the final version of the survey was implemented using the online platform Survio (www.survio.com). The poll was designed based on the concept that respondents were individually asked to evaluate the clinical relevance of each potential reporting element, classifying them as either ‘essential’ or ‘non-essential’. With support from the German Society of Neuroradiology (DGNR), German Society of Neurosurgery (DGNC), and German Society of Radiation Oncology (DEGRO), all registered specialists could be invited to take part in the survey via email between November 2022 and March 2023. Eligibility for participation in this nationwide project required board certification as well as regular practical experience in the imaging diagnosis and/or treatment of patients with intracranial meningiomas. Taken together, the study design builds principally on previous work in which the clinically essential MRI reporting categories for intracranial gliomas and sellar tumors were identified^15,16^. A synopsis of the course of study is presented in Fig. 1 ‘study course_meningioma mri reporting’.

### Study participants

A total of 150 medical specialists from the Federal Republic of Germany participated in the online survey during the period from November 2, 2022, to March 13, 2023. Among them were 46 radiologists with a specialization in neuroradiology (7.7% of 600 registered specialists in the DGNR), 60 neurosurgeons, 42 radiation oncologists, and 2 experts with combined specialization in neurosurgery and radiation therapy (3.9% of 1581 registered specialists in the DGNC/4.4% of 1004 registered specialists in the DEGRO). Table 1 ‘survey participation_meningioma mri reporting’ provides an overview of the relative numbers of respondents in relation to the respective membership numbers of the corresponding German medical societies of neuroradiology, neurosurgery, and radiation oncology. All respondents stated that they were involved in the clinical management of meningioma patients, either diagnostically or therapeutically, as part of their daily routine practice. Among them, 48.0% (72/150) were employed at institutions with an annual patient volume of ≥ 50 meningioma cases.

**Table 1:** Survey participation.

Medical society	DGNR	DGNC/BDNC	DEGRO	Significance
Specialty	Neuroradiology	Neurosurgery	Radiation oncology	
Survey participants	46	62	44	
Registered specialists	600	1581	1004	
Participation rate	7.7% (46/600)	3.9% (62/1581)	4.4% (44/1004)	0.0010*

Participation in the survey with regard to the various medical societies involved. The total exceeds the overall number of participants in the study (n = 150) because two responders are specialists in both neurosurgery and radiotherapy. DEGRO German Society of Radiation Oncology. DGNC/BDNC German Society of Neurosurgery/Professional Association of German Neurosurgeons. DGNR German Society of Neuroradiology.

*Chi-square test (significance level 0.05).

### Reporting items

The survey aimed to assess essential radiology reporting elements related to diagnostic meningioma imaging. These key categories included the location and extent of the meningioma, as well as its impact on neighboring anatomical structures such as brain parenchyma, dura mater, bones, blood vessels, and cranial nerves. The survey also focused on various internal characteristics of the tumor, such as contrast uptake, cysts, calcifications, hemorrhages, and necrosis. In addition to these primary elements, the experts evaluated signs of mass effect, the use of advanced MRI techniques like diffusion, perfusion, and proton spectroscopy, and treatment-related changes (e.g., extent of surgical resection). Finally, the participants were asked to assess the significance of meningioma-specific classifications in MRI reporting. These classifications included the Simpson classification (for resection extent), RANO criteria (for treatment response), Sindou classification (for venous sinus invasion) as well as Nauta and Zee classification (for associated cysts)^3,17–20^.

### Data presentation and statistics

Overall and group-specific survey results are reported descriptively in terms of fractions (approvals/total number of responses) and associated percentages. The different medical disciplines were compared with each other in terms of their levels of agreement with the potential MRI reporting elements under study using a two-sided Chi-square test or Fisher’s exact test, with a p value < 0.05 considered statistically significant. The comparison was performed in a two-step process: first, we contrasted clinicians (neurosurgeons and radiation oncologists combined) with neuroradiologists; next, we compared neurosurgeons with radiation oncologists. All statistical procedures were performed using GraphPad Prism version 10.0.1 for macOS (GraphPad Software, Boston, Massachusetts, USA; www.graphpad.com).

### Previous presentation

Parts of this study have been presented as an oral contribution at the 58th annual meeting of the German Society of Neuroradiology. The meeting was held in Kassel (Germany) from October 4 to 6, 2023. The meeting abstracts are available at https://doi.org/10.1007/s00062-023-01336-5.

## Results

### Cumulative rating of meningioma MRI reporting items

**Fig. 2:**
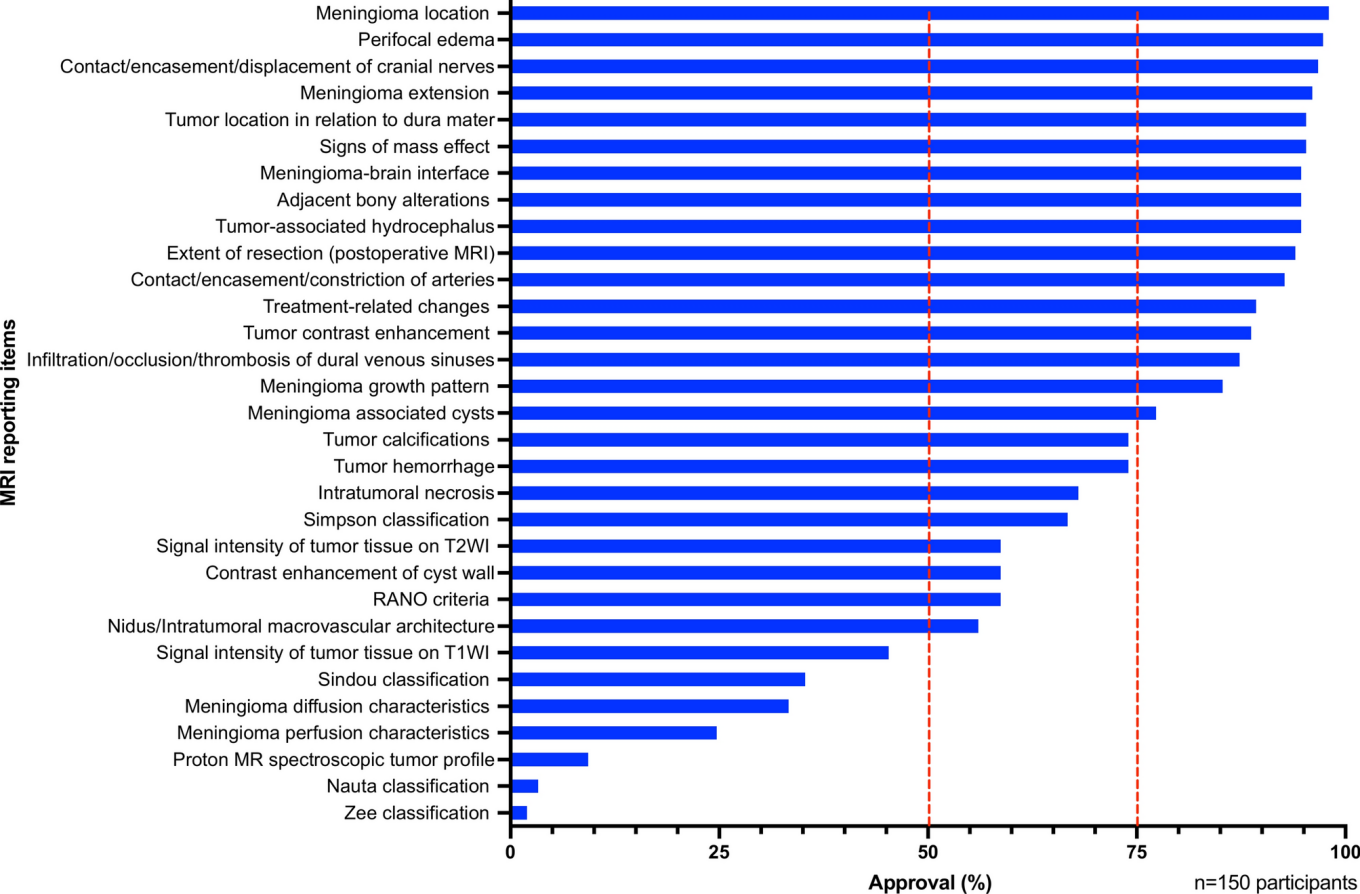
Cumulative assessment of MRI reporting items in meningioma imaging.

Over 75.0% of all participants rated the meningioma location, growth pattern, and extent with respect to the dura mater, adjacent brain tissue, and neighboring bony structures as essential elements of an MRI report. Additionally, for more than three-quarters of respondents, involvement of vascular structures and cranial nerves as well as potential tumor-related complications such as mass effect, occlusive hydrocephalus, and perifocal edema were crucial findings. The intrinsic meningioma characteristics that received the highest agreement (> 75.0%) were the contrast-enhancement behavior and the presence of any tumor-associated cysts. After treatment, more than three out of four experts voted for the inclusion of resection extent and treatment-related changes in the radiological report. Tumor features such as macrovascular architecture, hemorrhage, calcification, necrosis, contrast enhancement of cyst walls, and T2 signal intensity of the neoplastic tissue received somewhat lower support (50.0% to 75.0% of all respondents). While the Simpson classification of resection extent and the RANO criteria for therapy response were predominantly regarded as significant components of meningioma MRI reporting, most participants did not perceive a compelling need to employ other meningioma-specific classifications (i.e., Sindou, Nauta, Zee). Moreover, outcomes from advanced MRI techniques, such as diffusion, perfusion, and proton spectroscopy, were deemed dispensable for characterizing this tumor entity by the majority of surveyed experts. The precise approval ratings pertaining to these and all other evaluated potential meningioma MRI reporting items can be extracted from Fig. 2 ‘cumulative assessment of MRI reporting items in meningioma imaging’. Most respondents preferred a size description of meningiomas in the form of stating three dimensions as length times width times height (100/150; 66.7%), with a comparison desired both with the initial and immediately preceding MRI (126/150; 84.0%). The preferred intervals for the first follow-up imaging of presumably benign meningiomas were between 4 and 6 months (90/150; 60.0%), and further follow-ups were mostly considered appropriate after 7 to 12 months (102/150; 68.0%). According to the participants, postoperative MRI baseline examinations should be performed within 3 months after the resection (benign meningiomas 82/150; 54.7%/non-benign meningiomas 127/150; 84.7%), while a longer interval of more than 3 months until the first follow-up was favored after radiotherapy (80/150; 53.3%). More detailed information regarding the size description of meningiomas, preferred baseline examinations, and follow-up intervals can be found in Table 2 ‘tumor extension_baseline_follow up_meningioma mri reporting’.

**Table 2:** Cumulative and specialty-specific preferences regarding the size evaluation of meningiomas, the most suitable baseline MRI examination for growth determination, and appropriate follow-up intervals in the case of conservative, surgical, or radiotherapeutic management.

	All experts	Neurosurgeons + radiation oncologists	Neuroradiologists	P value
	Group 1/2/3	Group 1/2	Group 3	Group 1/2 vs. 3
Preferred measurement of meningioma extension				
Maximum extension in one dimension (one-dimensional)	6/150 (4.0)	1/104 (1.0)	5/46 (10.9)	**0.0005**
Length times width (two-dimensional)	10/150 (6.7)	5/104 (4.8)	5/46 (10.9)	
Cross-sectional area (two-dimensional)	1/150 (0.7)	1/104 (1.0)	0/46 (0.0)	
Length times width times height (three-dimensional)	100/150 (66.7)	66/104 (63.5)	34/46 (73.9)	
Volumetry (three-dimensional)	33/150 (22.0)	31/104 (29.8)	2/46 (4.3)	
Preferred baseline MRI for meningioma growth measurement				
First MRI examination	16/150 (10.7)	14/104 (13.5)	2/46 (4.3)	0.2203
Most recent MRI before the current examination	8/150 (5.3)	6/104 (5.8)	2/46 (4.3)	
First and most recent MRI before the current examination	126/150 (84.0)	84/104 (80.8)	42/46 (91.3)	
Preferred time intervals for follow-up MRI examinations				
First follow-up of presumed benign meningioma				
0–3 months	39/150 (26.0)	38/104 (36.5)	1/46 (2.2)	** < 0.0001**
4–6 months	90/150 (60.0)	57/104 (54.8)	33/46 (71.7)	
7 + months	21/150 (14.0)	9/104 (8.7)	12/46 (26.1)	
Further follow-up of presumed benign meningioma				
0–6 months	34/150 (22.7)	30/104 (28.8)	4/46 (8.7)	**0.0122**
7–12 months	102/150 (68.0)	67/104 (64.4)	35/46 (76.1)	
13 + months	14/150 (9.3)	7/104 (6.8)	7/46 (15.2)	
First postoperative MRI after resection of benign meningioma				
0–3 months	82/150 (54.7)	66/104 (63.5)	16/46 (34.8)	**0.0044**
4–6 months	54/150 (36.0)	31/104 (29.8)	23/46 (50.0)	
7 + months	14/150 (9.3)	7/104 (6.7)	7/46 (15.2)	
First postoperative MRI after resection of non-benign meningioma				
0–3 months	127/150 (84.7)	96/104 (92.3)	31/46 (67.4)	**0.0004**
4–6 months	22/150 (14.7)	8/104 (7.7)	14/46 (30.4)	
7 + months	1/150 (0.7)	0/104 (0.0)	1/46 (2.2)	
First MRI after radiotherapy of meningioma				
0–3 months	70/150 (46.7)	60/104 (57.7)	10/46 (21.7)	**0.0002**
4–6 months	75/150 (50.0)	41/104 (39.4)	34/46 (73.9)	
7 + months	5/150 (3.3)	3/104 (2.9)	2/46 (4.3)	

Agreement rates are given as fractions (n/N) and percentages (in parentheses). All P values were calculated using two-sided Chi-square test (significant results highlighted in bold; significance level 0.05).

### Priority differences between neuroradiologists and neurooncology clinicians

**Fig. 3:**
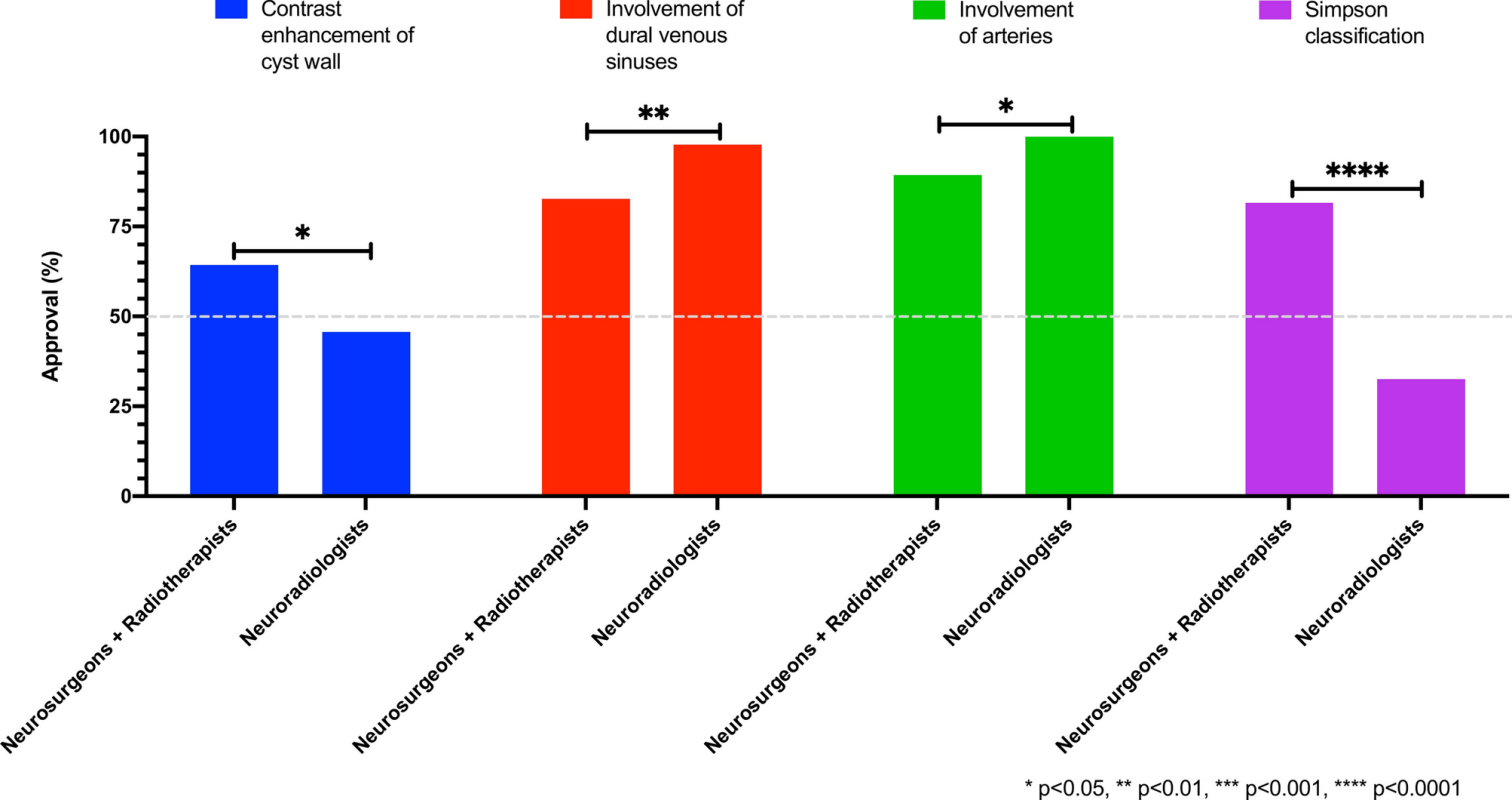
Meningioma MRI reporting items with significantly different assessments by neurosurgeons/radiation oncologists compared to neuroradiologists.

Neurosurgeons and radiation oncologists prioritized the implementation of the Simpson classification and contrast enhancement in tumor-associated cyst walls, while neuroradiologists valued the description of vessel involvement more than neurooncologists. A comprehensive depiction of these significantly divergent evaluated reporting categories (p < 0.05) is provided in Fig. 3 ‘comparison of significantly different items_neurosurgeons and radiation oncologists versus neuroradiologists’, while Table 3 ‘cumulative and discipline-specific assessment_meningioma mri reporting’ illustrates the distinct assessment of the remaining items by neurooncologists and neuroradiologists, respectively.

**Table 3:** Cumulative and discipline-specific expert assessment of potential reporting items for MRI examinations of meningioma patients.

Item	All experts	Neurosurgeons + radiation oncologists	Neurosurgeons	Radiation oncologists	Neuroradiologists	P value 1	P value 2
	Group 1/2/3	Group 1/2	Group 1	Group 2	Group 3	Group 1/2 vs. 3	Group 1 vs. 2
Meningioma location (epicenter)	147/150 (98.0)	101/104 (97.1)	59/60 (98.3)	40/42 (95.2)	46/46 (100.0)	0.5531	0.5670
Meningioma growth pattern (spherical/lobular/en plaque)	128/150 (85.3)	86/104 (82.7)	53/60 (88.3)	31/42 (73.8)	42/46 (91.3)	0.2152	0.0691
Meningioma extension (compartments)	144/150 (96.0)	98/104 (94.2)	57/60 (95.0)	39/42 (92.9)	46/46 (100.0)	0.1779	0.6882
Signal intensity of tumor tissue on T1WI (compared to grey matter)	68/150 (45.3)	51/104 (49.0)	21/60 (35.0)	29/42 (69.0)	17/46 (37.0)	0.2136	**0.0012**
Signal intensity of tumor tissue on T2WI (compared to grey matter)	88/150 (58.7)	58/104 (55.8)	36/60 (60.0)	20/42 (47.6)	30/46 (65.2)	0.3688	0.2322
Tumor contrast enhancement (intensity, homogeneity)	133/150 (88.7)	92/104 (88.5)	54/60 (90.0)	36/42 (85.7)	41/46 (89.1)	> 0.9999	0.5448
Meningioma diffusion characteristics (apparent diffusion coefficient)	50/150 (33.3)	33/104 (31.7)	17/60 (28.3)	14/42 (33.3)	17/46 (37.0)	0.5754	0.6638
Meningioma perfusion characteristics (relative cerebral blood volume)	37/150 (24.7)	29/104 (27.9)	19/60 (31.7)	8/42 (19.0)	8/46 (17.4)	0.2188	0.1777
Proton MR spectroscopic tumor profile	14/150 (9.3)	13/104 (12.5)	5/60 (8.3)	7/42 (16.7)	1/46 (2.2)	0.0648	0.2248
Nidus/Intratumoral macrovascular architecture (MR angiography)	84/150 (56.0)	56/104 (53.8)	48/60 (80.0)	7/42 (16.7)	28/46 (60.9)	0.4780	** < 0.0001**
Tumor calcifications (T2*/susceptibility weighted imaging)	111/150 (74.0)	73/104 (70.2)	52/60 (86.7)	20/42 (47.6)	38/46 (82.6)	0.1570	** < 0.0001**
Intratumoral necrosis	102/150 (68.0)	67/104 (64.4)	40/60 (66.7)	27/42 (64.3)	35/46 (76.1)	0.1865	0.8346
Tumor hemorrhage	111/150 (74.0)	75/104 (72.1)	46/60 (76.7)	27/42 (64.3)	36/46 (78.3)	0.5456	0.1881
Meningioma associated cysts	116/150 (77.3)	76/104 (73.1)	42/60 (70.0)	33/42 (78.6)	40/46 (87.0)	0.0894	0.3703
Contrast enhancement of cyst wall	88/150 (58.7)	67/104 (64.4)	32/60 (53.3)	34/42 (81.0)	21/46 (45.7)	**0.0473**	**0.0059**
Meningioma-brain interface (circumscript/invasive)	142/150 (94.7)	97/104 (93.3)	57/60 (95.0)	39/42 (92.9)	45/46 (97.8)	0.4357	0.6882
Tumor location in relation to dura mater	143/150 (95.3)	100/104 (96.2)	58/60 (96.7)	40/42 (95.2)	43/46 (93.5)	0.6764	> 0.9999
Infiltration/occlusion/thrombosis of dural venous sinuses	131/150 (87.3)	86/104 (82.7)	58/60 (96.7)	27/42 (64.3)	45/46 (97.8)	**0.0079**	** < 0.0001**
Perifocal edema	146/150 (97.3)	100/104 (96.2)	59/60 (98.3)	39/42 (92.9)	46/46 (100.0)	0.3129	0.3032
Adjacent bony alterations (hyperostosis/osteolysis)	142/150 (94.7)	97/104 (93.3)	57/60 (95.0)	39/42 (92.9)	45/46 (97.8)	0.4357	0.6882
Contact/encasement/constriction of arteries	139/150 (92.7)	93/104 (89.4)	60/60 (100.0)	32/42 (76.2)	46/46 (100.0)	**0.0186**	** < 0.0001**
Contact/encasement/displacement of cranial nerves	145/150 (96.7)	100/104 (96.2)	60/60 (100.0)	38/42 (90.5)	45/46 (97.8)	> 0.9999	**0.0263**
Tumor-associated hydrocephalus	142/150 (94.7)	98/104 (94.2)	59/60 (98.3)	37/42 (88.1)	44/46 (95.7)	> 0.9999	0.0790
Signs of mass effect (midline shift/ventricle compression/herniation)	143/150 (95.3)	98/104 (94.2)	56/60 (93.3)	40/42 (95.2)	45/46 (97.8)	0.6764	> 0.9999
Extent of resection (postoperative MRI)	141/150 (94.0)	98/104 (94.2)	55/60 (91.7)	41/42 (97.6)	43/46 (93.5)	> 0.9999	0.3965
Treatment-related changes (resection cavity/gliosis/postradiogenic leukoencephalopathy)	134/150 (89.3)	91/104 (87.5)	48/60 (80.0)	41/42 (97.6)	43/46 (93.5)	0.3926	**0.0131**
Nauta classification (meningioma cysts)	5/150 (3.3)	4/104 (3.8)	2/60 (3.3)	2/42 (4.8)	1/46 (2.2)	> 0.9999	> 0.9999
Zee classification (meningioma cysts)	3/150 (2.0)	2/104 (1.9)	1/60 (1.7)	1/42 (2.4)	1/46 (2.2)	> 0.9999	> 0.9999
Sindou classification (infiltration of superior sagittal sinus)	53/150 (35.3)	42/104 (40.4)	32/60 (53.3)	8/42 (19.0)	11/46 (23.9)	0.0642	**0.0005**
Simpson classification (extent of resection)	100//150 (66.7)	85/104 (81.7)	54/60 (90.0)	29/42 (69.0)	15/46 (32.6)	** < 0.0001**	**0.0100**
RANO criteria (treatment response)	88/150 (58.7)	64/104 (61.5)	32/60 (53.3)	30/42 (71.4)	24/46 (52.2)	0.2881	0.0987

Agreement rates regarding integration of each item into MRI reports are given as fractions (n/N) and percentages (in parentheses). The combined group of neurosurgeons and radiotherapists (group 1/2) contains two more participants than the groups 1 and 2 considered individually because two respondents were specialists in both neurosurgery and radiotherapy and were therefore included only in the combined cohort. All P values were calculated using two-sided Fisher’s exact test (significant results highlighted in bold; significance level 0.05).

### Preference variations between neurosurgeons and radiation oncologists

**Fig. 4:**
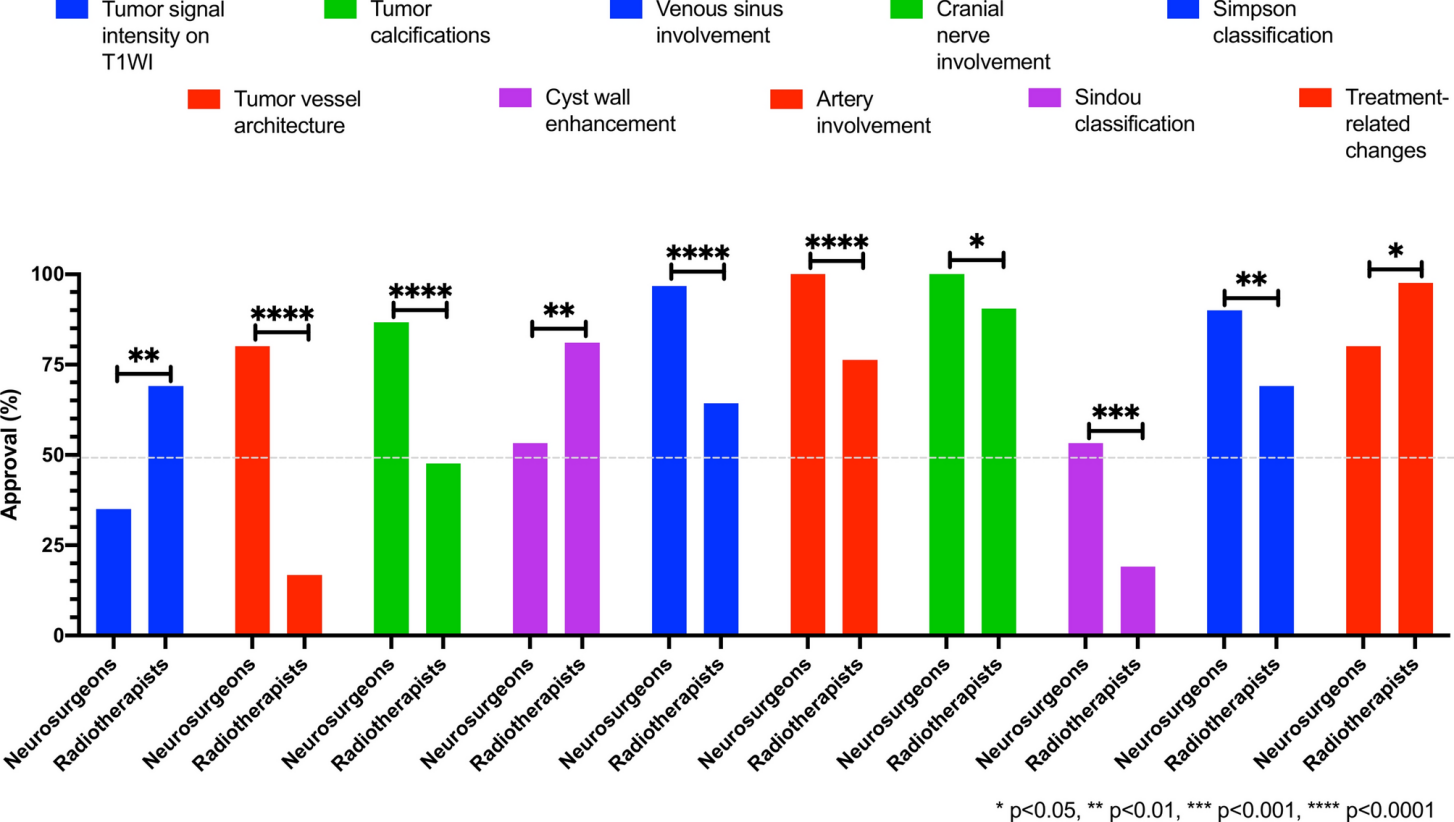
Meningioma MRI reporting items with significantly different assessments by neurosurgeons compared to radiation oncologists.

In comparison to radiation oncologists, neurosurgeons exhibited a greater interest in describing specific characteristics of tumor internal structure (vessel architecture, calcifications), incorporating involvement of brain-feeding arterial and venous vessels as well as cranial nerves, and applying the Sindou and Simpson classifications. Conversely, radiation oncologists attributed greater significance to the T1 signal intensity of meningiomas, contrast enhancement of cyst walls, and treatment-related changes. Figure 4 ‘comparison of significantly different items_neurosurgeons versus radiation oncologists’ delineates these significant assessment differences (p < 0.05) between neurosurgeons and radiation oncologists. A comprehensive breakdown of all other reporting categories can be found in Table 3 ‘cumulative and discipline-specific assessment_meningioma mri reporting’.

## Discussion

### Integration of clinical expert information needs and context-specific structured reporting

The present study, involving a total of 150 participating experts, constitutes the first nationwide comprehensive survey to date regarding the content requirements of MRI reports for intracranial meningiomas by the representatives of the treating clinical disciplines. It compares these findings with the contemporary prevailing perspective of the neuroradiological community. The insights gained from the clinicians’ information preferences may serve as a starting point for the development of a context-specific structured reporting template for MRI examinations of meningioma patients. Context-specific radiological reporting represents an advancement of structured reporting tailored specifically to certain diseases or examination indications. In the neuroradiological context, this approach has already shown a clear preference over traditional narrative reporting for numerous other conditions, such as vestibular schwannoma and glioma. This benefit seems to be attributable, among other factors, to the improved understandability of reported findings and impressions, as well as the more comprehensive coverage of relevant content^21,22^. This hypothesis is supported by an analysis by Boll et al., who, using the “voice-of-the-customer method” at their institution, demonstrated that the most significant deficiency perceived by clinicians in traditional radiological reporting is the inadequate consideration of their specific practice-relevant information needs^23^.

### Key elements of meningioma MRI reporting from a clinical perspective

The radiology reporting elements deemed most important in meningioma imaging by the surveyed experts can be generally categorized into four main groups: (1) Tumor localization, growth pattern, and extent; (2) structural characteristics of the mass such as contrast enhancement and associated cyst formation; (3) tumor-related complications like involvement/impairment of adjacent anatomical structures (brain tissue + / − perifocal edema, blood vessels, and cranial nerves), mass effect, and occlusive hydrocephalus; and (4) treatment-related changes including the extent of resection. This assessment may serve as a pragmatic basic guidance for the findings section of the MRI report. To this end, we have consolidated the items most endorsed by the surveyed experts (> 50% agreement) into a clinician-oriented reporting template (refer to Supplement ‘structured mri reporting template for intracranial meningiomas’). It broadly aligns with the proposed interpretation checklist of the living reference work of the European Society of Neuroradiology. In this checklist, all above-mentioned elements from the four categories are listed, with the exception of meningeoma-associated cysts^5^. Remarkably, recent findings have indicated an increased incidence of WHO Grade 2 meningiomas in cases involving associated tumor cysts, suggesting a compelling reason for giving special attention to this diagnostic element^24^. The mentioned factors, tumor size, location, and involvement of neighboring neurovascular structures, are of paramount clinical importance as they are essential determinants of surgical risk, achievable extent of resection, and consequently, the likelihood of meningioma recurrence^2,25–28^. For these factors, it has also been demonstrated that in the case of asymptomatic meningiomas, they constitute a substantial basis for determining a treatment strategy and are accorded greater significance compared to demographic factors^29^. Moreover, in larger observational studies, a correlation has been established between the characteristics of meningioma size and location with their growth behavior. A larger tumor diameter and a position away from the skull base were associated with progression^30,31^. Benign meningiomas located at the skull base also appear to be less prone to malignant transformation in comparison to counterparts located in other intracranial sites^32^. Following neurosurgery, the extent of resection warrants special attention, as it represents the only currently known modifiable risk factor for meningioma recurrence. Patients undergoing non-Simpson Grade I resection, as opposed to complete removal of the meningioma, exhibit a significantly increased risk of tumor progression or recurrence^2,33^. As a result, the majority of surveyed experts considered the Simpson grading system, which assesses the extent of resection and the associated risk of symptomatic tumor recurrence, as a crucial scale in meningioma evaluation, alongside the established RANO criteria for treatment response^17^. However, it must be noted that the Simpson scale, which was developed in an earlier era before the availability of modern imaging techniques such as CT and MRI, has certain limitations. It originated from the neurosurgical perspective, not neuroradiological, relying on intraoperative visual assessment of resection. Thus, it is subject to the surgeon’s individual interpretation and numerous potential inaccuracies, including restrictions due to limited surgical access^34^. In a comparative study, the neurosurgical assessment of resection extent using the Simpson scale was compared with a modified MRI-based grading system (Meningioma Group Amsterdam (MEGA) Grading System of Meningioma Removal), where differentiation was made only between removal and preservation of the dura mater, without considering radiologically unidentifiable pachymeningeal coagulation (corresponding to Simpson Grade II). The study showed good agreement between the two methods^35^. While the prognostic value of the extent of resection is generally undisputed, it should be noted that despite the widespread use of Simpson grading, clinical studies often adopt a simpler dichotomous classification of gross total resection (i.e., no residual solid tumor) versus subtotal resection^2^. However, investigations concerning patients with subtotal meningioma resection suggest that minimizing the residual volume to less than 4–5 cm^3^ (i.e., amenable to postoperative stereotactic radiosurgery) may be associated with improved recurrence free survival, underscoring the importance of tumor volumetry^36^.

### Significance of advanced MRI techniques

The neurosurgical, radiotherapeutic, and neuroradiological experts participating in the study predominantly considered the advanced MRI techniques of diffusion, perfusion, and proton spectroscopy as non-essential within the scope of clinical meningioma imaging. This stance is supported by clinical experience, indicating that conventional anatomical imaging is typically qualitatively sufficient to distinguish intracranial meningiomas from other neoplastic entities^28^. However, in ambiguous cases, the aforementioned methods can partially contribute to confirming the diagnosis^37^. Prior studies conducted on MRI diffusion have revealed a highly heterogeneous picture regarding the value of this technique in determining the malignancy grade, rendering the use of the apparent diffusion coefficient (ADC) generally not recommended for therapeutic management^37–40^. On the other hand, the new 2021 WHO classification of tumors of the CNS primarily grades meningiomas based on molecular factors (including homozygous deletion of CDKN2A/B and TERT promoter mutation) and histological features (such as mitotic figures, cellularity, nuclear-to-cytoplasmic ratio, brain invasion, and necrosis)^41^. These histological characteristics lead to significant changes in the MRI appearance of the tumors. Notably, a recent meta-analysis demonstrated a significant correlation between the abovementioned features including diffusion restriction, an established surrogate marker of increased cellularity, and the pathological grade of meningiomas^42^. Proton spectroscopy shows, in addition to the elevated choline and decreased NAA values typical for many tumors in the CNS, an increase in alanine and an additional metabolite peak at 3.8 parts per million, characteristic of meningiomas^37,43,44^. Also, for MR perfusion, which reflects the degree of vascularization of a lesion, it has been demonstrated that particularly the relative cerebral blood volume in the peritumoral edema can assist in the preoperative differentiation between benign and malignant meningiomas^37,45^.

### Preferred description of tumor size

The study participants preferred the three-dimensional description of tumor size in terms of length times width times height over one- or two-dimensional measurements as implemented in the Response Evaluation Criteria In Solid Tumors (RECIST; one-dimensional) and RANO criteria (two-dimensional)^46^. The preference for three-dimensional data appears rational, given that in a recent study conducted on a large cohort of patients with recurrent meningiomas under pharmacological therapy, stronger associations with overall survival were observed for volumetric progression criteria^47^. Additionally, in the recently updated RANO 2.0 criteria for gliomas, volumetric data are mentioned at least as an alternative to the classical recommended two-dimensional measurement of tumor extent^8^. However, to date, there is still a lack of evidence demonstrating that the clinical benefit of tumor volumetry outweighs the associated increased effort due to technical challenges and the additional time required. Therefore, as a provisional compromise, a simpler size measurement using meningioma diameter in three spatial dimensions appears to be a practical approach.

### Preferred imaging follow-up intervals

In our survey, most experts favored an initial MRI follow-up for incidentally discovered benign appearing meningiomas after 4–6 months. According to the EANO guideline on the diagnosis and management of meningiomas, an annual surveillance scan is recommended for the first 5 years, based on the lowest level of evidence (Class 4)^2^. This recommendation has been implemented in practice at least in some centers^28^. On the other hand, a national survey conducted by the Guideline Working Group of the Korean Society for Neuro-Oncology revealed a highly heterogeneous landscape concerning radiological follow-up plans without consensus. Consistent with our findings, a substantial portion of surveyed Korean experts advocated for an earlier imaging assessment after just 6 months^29^. In contrast to rigidly defined collective MRI control intervals, Islim and colleagues developed an online freely accessible calculator (www.impact-meningioma.com) based on well-known progression-relevant imaging characteristics (tumor T2/FLAIR signal intensity, meningioma volume, proximity to critical neurovascular structures, and peritumoral signal change) in conjunction with patient-specific factors (age, comorbidity, and performance status) to aid in determining the control interval on an individual basis^48^. Concerns regarding gadolinium deposition in brain tissue in the case of frequent or close MRI monitoring could potentially be addressed by refraining from contrast agent administration in at least a subset of these examinations. A comparative study has already demonstrated a strong correlation between meningioma size determination utilizing pre-contrast T2-weighted and post-contrast T1-weighted images^7^. The surveyed experts preferred a postoperative MRI within 3 months after meningioma surgery. Although the ideal timing has not been defined so far by clinical studies, Slot et al. recommend a 3 month follow-up to avoid overestimating residual tumor tissue due to early postoperative changes^35^.

### Content priorities by medical specialty

In comparison to neuroradiologists, representatives from therapeutic disciplines assigned greater importance to Simpson Grading and contrast agent uptake in cysts. The relatively lower affinity of neuroradiologists for the Simpson Classification may be primarily attributed to its subjective nature, as it relies on the perspective of the operating neurosurgeon and includes factors that cannot be adequately assessed radiologically. However, this problem could be circumvented by applying the newer MRI-based Meningioma Group Amsterdam (MEGA) Grading System. This classification consolidates the original Simpson Grades II and III due to the absence of a radiological equivalent for endothermic dura coagulation. Good agreement has already been demonstrated for both classifications, as mentioned above^35^. Taking into account the contrast uptake in cyst walls seems rational, allowing differentiation between tumor cell-containing (enhancing) and gliotic (non-enhancing) cyst walls^19^. Nevertheless, the clinical benefit of total cyst wall resection has not been definitively proven so far^24^. In direct comparison, neurosurgeons demonstrated a greater interest in the involvement of blood vessels and cranial nerves, whereas radiation oncologists, in turn, emphasized treatment-associated changes. These differing focuses vividly illustrate, on one hand, the significance of the tumor’s spatial relationship with neurovascular structures for the surgical strategy, and on the other hand, the multiple potential sequelae that can occur over time due to brain parenchyma irradiation, despite continuous technological advances (e.g., radiation-induced secondary tumors, leukoencephalopathy, radiation-induced vasculopathy/Moyamoya syndrome)^28,49^.

### Strengths and limitations

This study constitutes the first nationwide survey addressing the definition of content requirements for MRI reports of intracranial meningiomas, taking into account the perspectives of treating physicians (neurosurgeons and radiation oncologists) as well as diagnostic experts (neuroradiologists). Despite a substantial number of participants in the study (n = 150) and a relatively balanced representation across involved clinical disciplines, the proportion, approximately 4–8% relative to the total membership of respective medical professional societies, remains low compared to the spectrum of published survey studies, introducing a potential response bias^50^. On the other hand, web surveys are generally known to have a significantly lower participation rate compared to other polling techniques^51^. It is important to note, however, that regular therapeutic or diagnostic interaction with meningioma patients was a prerequisite for participation, limiting eligibility to a specific subset of society members. The aforementioned participation rate corresponds to a Response Rate Type 1 according to the current standard definitions provided by AAPOR (American Association for Public Opinion Research; https://aapor.org), also known as the minimum response rate, which is defined as the number of complete interviews divided by the number of interviews (complete plus partial) plus the number of non-interviews (refusals and break-offs, plus non-contacts, plus others) plus all cases of unknown eligibility. For further calculations, an additional inquiry to the German Society of Neurosurgery was conducted to determine the work environment of the surveyed specialists as a surrogate marker for the spectrum of professional activity. At the time of the online survey, 74 out of a total of 1,581 registered German specialists were in private practice. Assuming, with reasonable certainty, that no brain surgeries are performed in private practices due to the lack of availability of intensive care beds (i.e., e = 0% estimated proportion of cases that are eligible), and considering that nearly all neurosurgeons working in hospitals in Germany are involved in the management of meningioma patients to a greater or lesser extent due to the high prevalence of this condition (i.e., e = 100% estimated proportion of cases that are eligible), a conservative estimate of the survey response rate type 3 (RR3) can be made. This calculation results in a RR3 of 4.1%. Based on our experience, non-hospital-affiliated (i.e., privately practicing) neuroradiologists and radiation oncologists frequently manage meningioma patients due to the widespread prevalence of this condition. Therefore, in our opinion, these professionals should not be classified as ineligible to participate. In principle, the design of a preconfigured questionnaire with a defined set of items raises the possibility of incomplete thematic coverage. We attempted to prevent this potential bias by giving all participants the opportunity at the end of the survey to submit their own supplementary requests in free-text format. The primary intention of the study is to provide a recommendation as to what should be included in the MRI report according to the “voice-of-the-customer method” rather than suggest what should be left out to ensure that all contextual clinical information needs are met. In this context, it must be pointed out that additional image information, which may not be considered essential by the referring physicians, may still be important for the interpreting radiologist to finally make the correct differential diagnosis. The present work is explicitly limited to MRI as the current gold standard in the imaging of meningiomas. For this reason, no direct conclusions should be drawn from it regarding other potentially relevant diagnostic modalities, such as computed tomography or positron emission tomography. Furthermore, we spatially restricted the project to intracranial meningiomas, so that no direct conclusions may be drawn concerning spinal meningiomas.

## Conclusion

The radiological report serves as a central communication tool in the interdisciplinary care of patients with intracranial meningiomas. To optimally address the informational needs of the treating clinical specialists, the reporting radiologist should prioritize the description of meningioma location and extent, growth pattern, tumor internal characteristics, impact on adjacent anatomical structures, mass effect, and tumor-associated hydrocephalus. For post-therapeutic follow-up examinations, the extent of resection and treatment-related changes are also of essential importance. Besides these fundamental requirements, additional features might be relevant for the radiologist to make the correct differential diagnosis. Consistent alignment with the clinical information needs of the neuro-oncology team has the potential for a sustainable improvement in interdisciplinary communication and, consequently, the care of patients with intracranial meningiomas.

## Supplementary Information

Supplementary Information 1.

Supplementary Information 2.

## Data Availability

All relevant information collected in this study is included in the manuscript or in the tables and figures. Metadata are available from the corresponding author upon reasonable request.

## References

[1] Ostrom, Q. T. et al. CBTRUS statistical report: Primary brain and other central nervous system tumors diagnosed in the United States in 2015–2019. *Neuro-Oncol.* **24**, v1–v95. 10.1093/neuonc/noac202 (2022). 36196752 10.1093/neuonc/noac202 PMC9533228

[2] Goldbrunner, R. et al. EANO guideline on the diagnosis and management of meningiomas. *Neuro-Oncol.* **23**, 1821–1834. 10.1093/neuonc/noab150 (2021). 34181733 10.1093/neuonc/noab150 PMC8563316

[3] Huang, R. Y. et al. Proposed response assessment and endpoints for meningioma clinical trials: report from the response assessment in neuro-oncology working group. *Neuro-Oncol.* **21**, 26–36. 10.1093/neuonc/noy137 (2019). 30137421 10.1093/neuonc/noy137 PMC6303427

[4] Ellingson, B. M. et al. Consensus recommendations for a standardized brain tumor imaging protocol in clinical trials. *Neuro Oncol.* **17**, 1188–1198. 10.1093/neuonc/nov095 (2015). 26250565 10.1093/neuonc/nov095 PMC4588759

[5] Thust, S. & Kumar, A. Extra-axial tumors. In *Clinical Neuroradiology* (eds Barkhof, F. et al.) 1–37 (Springer International Publishing, 2019).

[6] Starekova, J., Pirasteh, A. & Reeder, S. B. Update on gadolinium-based contrast agent safety, from the *AJR* special series on contrast media. *Am. J. Roentgenol.* **223**, e2330036. 10.2214/AJR.23.30036 (2024). 37850581 10.2214/AJR.23.30036

[7] Rahatli, F. K. et al. Can unenhanced brain magnetic resonance imaging be used in routine follow up of meningiomas to avoid gadolinium deposition in brain?. *Clin. Imaging* **53**, 155–161. 10.1016/j.clinimag.2018.10.014 (2019). 30343167 10.1016/j.clinimag.2018.10.014

[8] Wen, P. Y. et al. RANO 20: Update to the response assessment in neuro-oncology criteria for high- and low-grade gliomas in adults. *J. Clin. Oncol.*10.1200/JCO.23.01059 (2023). 37774317 10.1200/JCO.23.01059 PMC10860967

[9] Watts, J. et al. Magnetic resonance imaging of meningiomas: a pictorial review. *Insights Imaging* **5**, 113–122. 10.1007/s13244-013-0302-4 (2014). 24399610 10.1007/s13244-013-0302-4 PMC3948902

[10] Kunimatsu, A. et al. Variants of meningiomas: a review of imaging findings and clinical features. *Jpn. J. Radiol.* **34**, 459–469. 10.1007/s11604-016-0550-6 (2016). 27138052 10.1007/s11604-016-0550-6

[11] Krishnan, V., Mittal, M. & Sinha, M. Imaging spectrum of meningiomas: a review of uncommon imaging appearances and their histopathological and prognostic significance. *Pol. J. Radiol.* **84**, 630–653. 10.5114/pjr.2019.92421 (2019). 10.5114/pjr.2019.92421 PMC7016363 32082462

[12] Osborn, A. G. *Essentials of Osborn’s brain: a fundamental guide for residents and fellows* (Elsevier, 2020).

[13] Maggio, I. et al. Meningioma: not always a benign tumor. A review of advances in the treatment of meningiomas. *CNS Oncol.*10.2217/cns-2021-0003 (2021). 34015955 10.2217/cns-2021-0003 PMC8162186

[14] Kelley, K. Good practice in the conduct and reporting of survey research. *Int. J. Qual. Health Care* **15**, 261–266. 10.1093/intqhc/mzg031 (2003). 12803354 10.1093/intqhc/mzg031

[15] Huckhagel, T. & Riedel, C. MRT-befundung hirneigener tumoren. *Die Radiol.*10.1007/s00117-022-01014-6 (2022). 10.1007/s00117-022-01014-6 PMC9343312 35913575

[16] Huckhagel, T., Riedel, C., Flitsch, J. & Rotermund, R. What to report in sellar tumor MRI? A nationwide survey among German pituitary surgeons, radiation oncologists, and endocrinologists. *Neuroradiology*10.1007/s00234-023-03222-w (2023). 37735221 10.1007/s00234-023-03222-w PMC10567906

[17] Simpson, D. The recurrence of intracranial meningiomas after surgical treatment. *J. Neurol. Neurosurg. Psychiatry* **20**, 22–39. 10.1136/jnnp.20.1.22 (1957). 13406590 10.1136/jnnp.20.1.22 PMC497230

[18] Nauta, H. J. et al. Xanthochromic cysts associated with meningioma. *J. Neurol. Neurosurg. Psychiatry* **42**, 529–535. 10.1136/jnnp.42.6.529 (1979). 469560 10.1136/jnnp.42.6.529 PMC490257

[19] Zee, C. S. et al. Magnetic resonance imaging of cystic meningiomas and its surgical implications. *Neurosurgery* **36**, 482–488. 10.1227/00006123-199503000-00006 (1995). 7753347 10.1227/00006123-199503000-00006

[20] Sindou, M. P. & Alvernia, J. E. Results of attempted radical tumor removal and venous repair in 100 consecutive meningiomas involving the major dural sinuses. *JNS* **105**, 514–525. 10.3171/jns.2006.105.4.514 (2006). 10.3171/jns.2006.105.4.514 17044551

[21] Mamlouk, M. D., Chang, P. C. & Saket, R. R. Contextual radiology reporting: A new approach to neuroradiology structured templates. *ANJR Am. J. Neuroradiol.*10.3174/ajnr.A5697 (2018). 10.3174/ajnr.A5697 PMC7410548 29903922

[22] Gore, A. et al. Institutional implementation of a structured reporting system: Our experience with the brain tumor reporting and data system. *Acad. Radiol.* **26**, 974–980. 10.1016/j.acra.2018.12.023 (2019). 30661977 10.1016/j.acra.2018.12.023

[23] Boll, D. T., Rubin, G. D., Heye, T. & Pierce, L. J. Affinity chart analysis: A method for structured collection, aggregation, and response to customer needs in radiology. *Am. J. Roentgenol.* **208**, W134–W145. 10.2214/AJR.16.16673 (2017). 28140618 10.2214/AJR.16.16673

[24] Go, K. et al. Cystic meningiomas: Correlation between radiologic and histopathologic features. *Brain Tumor Res. Treat.*10.14791/btrt.2018.6.e3 (2018). 29644810 10.14791/btrt.2018.6.e3 PMC5932295

[25] Adachi, K. et al. ABC surgical risk scale for skull base meningioma: a new scoring system for predicting the extent of tumor removal and neurological outcome: Clinical article. *JNS* **111**, 1053–1061. 10.3171/2007.11.17446 (2009). 10.3171/2007.11.17446 19119879

[26] Scheitzach, J., Schebesch, K.-M., Brawanski, A. & Proescholdt, M. A. Skull base meningiomas: neurological outcome after microsurgical resection. *J. Neurooncol.* **116**, 381–386. 10.1007/s11060-013-1309-x (2014). 24257965 10.1007/s11060-013-1309-x

[27] May, M. et al. Role of risk factors, scoring systems, and prognostic models in predicting the functional outcome in meningioma surgery: multicentric study of 552 skull base meningiomas. *Neurosurg. Rev.* **46**, 124. 10.1007/s10143-023-02004-5 (2023). 37219634 10.1007/s10143-023-02004-5 PMC10205827

[28] Mehdorn, H. M. Intracranial meningiomas: A 30-year experience and literature review. In *Advances and Technical Standards in Neurosurgery* (ed. Schramm, J.) 139–184 (Springer International Publishing, 2016). 10.1007/978-3-319-21359-0_6 26508409

[29] Kim, S. K. et al. A national consensus survey for current practice in brain tumor management II: Diffuse midline glioma and meningioma. *Brain Tumor Res. Treat.*10.14791/btrt.2020.8.e6 (2020). 32390349 10.14791/btrt.2020.8.e6 PMC7221470

[30] Hashimoto, N. et al. Slower growth of skull base meningiomas compared with non–skull base meningiomas based on volumetric and biological studies: Clinical article. *JNS* **116**, 574–580. 10.3171/2011.11.JNS11999 (2012). 10.3171/2011.11.JNS11999 22175721

[31] Oya, S., Kim, S.-H., Sade, B. & Lee, J. H. The natural history of intracranial meningiomas. *J. Neurosurg.* **114**, 1250–1256. 10.3171/2010.12.JNS101623 (2011). 21250802 10.3171/2010.12.JNS101623

[32] Nakasu, S., Notsu, A., Na, K. & Nakasu, Y. Malignant transformation of WHO grade I meningiomas after surgery or radiosurgery: systematic review and meta-analysis of observational studies. *Neuro-Oncol. Adv.*10.1093/noajnl/vdaa129 (2020). 10.1093/noajnl/vdaa129 PMC7712809 33305267

[33] Hwang, W. L. et al. Imaging and extent of surgical resection predict risk of meningioma recurrence better than WHO histopathological grade. *Neuro Oncol.* **18**, 863–872. 10.1093/neuonc/nov285 (2016). 26597949 10.1093/neuonc/nov285 PMC4864259

[34] Schwartz, T. H. & McDermott, M. W. The Simpson grade: abandon the scale but preserve the message. *J. Neurosurg.*10.3171/2020.6.JNS201904 (2020). 33035995 10.3171/2020.6.JNS201904

[35] Slot, K. M. et al. Agreement between extent of meningioma resection based on surgical simpson grade and based on postoperative magnetic resonance imaging findings. *World Neurosurg.* **111**, e856–e862. 10.1016/j.wneu.2017.12.178 (2018). 29325959 10.1016/j.wneu.2017.12.178

[36] Chotai, S. & Schwartz, T. H. The Simpson grading: Is it still valid?. *Cancers* **14**, 2007. 10.3390/cancers14082007 (2022). 35454912 10.3390/cancers14082007 PMC9031418

[37] Tamrazi, B., Shiroishi, M. S. & Liu, C.-S.J. Advanced imaging of intracranial meningiomas. *Neurosurg. Clin. N. Am.* **27**, 137–143. 10.1016/j.nec.2015.11.004 (2016). 27012378 10.1016/j.nec.2015.11.004 PMC4936906

[38] Hakyemez, B. et al. The contribution of diffusion-weighted MR imaging to distinguishing typical from atypical meningiomas. *Neuroradiology* **48**, 513–520. 10.1007/s00234-006-0094-z (2006). 16786348 10.1007/s00234-006-0094-z

[39] Santelli, L. et al. Diffusion-weighted imaging does not predict histological grading in meningiomas. *Acta Neurochir* **152**, 1315–1319. 10.1007/s00701-010-0657-y (2010). 20428902 10.1007/s00701-010-0657-y

[40] Surov, A. et al. Use of diffusion weighted imaging in differentiating between maligant and benign meningiomas: A multicenter analysis. *World Neurosurg.* **88**, 598–602. 10.1016/j.wneu.2015.10.049 (2016). 26529294 10.1016/j.wneu.2015.10.049

[41] Louis, D. N. et al. The 2021 WHO classification of tumors of the central nervous system: a summary. *Neuro-Oncol.* **23**, 1231–1251. 10.1093/neuonc/noab106 (2021). 34185076 10.1093/neuonc/noab106 PMC8328013

[42] Upreti, T. et al. Meningioma grading via diagnostic imaging: A systematic review and meta-analysis. *Neuroradiology*10.1007/s00234-024-03404-0 (2024). 38902484 10.1007/s00234-024-03404-0 PMC11246317

[43] Matsusue, E. et al. Utility of 3T single-voxel proton MR spectroscopy for differentiating intracranial meningiomas from intracranial enhanced mass lesions. *Acta Radiol. Open* **10**, 205846012110094. 10.1177/20584601211009472 (2021). 10.1177/20584601211009472 PMC8215334 34211737

[44] De Stefano, F. A. et al. Unique magnetic resonance spectroscopy profile of intracranial meningiomas compared to gliomas: a systematic review. *Acta Neurol. Belg.*10.1007/s13760-022-02169-8 (2023). 36595196 10.1007/s13760-022-02169-8

[45] Zhang, H. et al. Perfusion MR imaging for differentiation of benign and malignant meningiomas. *Neuroradiology* **50**, 525–530. 10.1007/s00234-008-0373-y (2008). 18379768 10.1007/s00234-008-0373-y PMC2440923

[46] Chukwueke, U. N. & Wen, P. Y. Use of the response assessment in neuro-oncology (RANO) criteria in clinical trials and clinical practice. *CNS Oncol.*10.2217/cns-2018-0007 (2019). 30806082 10.2217/cns-2018-0007 PMC6499019

[47] Huang, R. Y. et al. Response assessment of meningioma: 1D, 2D, and volumetric criteria for treatment response and tumor progression. *Neuro-Oncol.*10.1093/neuonc/noy126 (2018). 30085283 10.1093/neuonc/noy126 PMC6374755

[48] Islim, A. I. et al. A prognostic model to personalize monitoring regimes for patients with incidental asymptomatic meningiomas. *Neuro-Oncol.* **22**, 278–289. 10.1093/neuonc/noz160 (2020). 31603516 10.1093/neuonc/noz160 PMC7032634

[49] Carr, C. M. et al. Intracranial long-term complications of radiation therapy: an image-based review. *Neuroradiology* **63**, 471–482. 10.1007/s00234-020-02621-7 (2021). 33392738 10.1007/s00234-020-02621-7

[50] Phillips, A. W. et al. Surveys of health professions trainees: Prevalence, response rates, and predictive factors to guide researchers. *Acad. Med.* **92**, 222–228. 10.1097/ACM.0000000000001334 (2017). 27532869 10.1097/ACM.0000000000001334

[51] Daikeler, J., Bošnjak, M. & Lozar Manfreda, K. Web versus other survey modes: An updated and extended meta-analysis comparing response rates. *J. Survey Stat. Methodol.* **8**, 513–539. 10.1093/jssam/smz008 (2020).

