# Supplementary material for: Identification of key elements in MRI reporting of intracranial meningiomas based on a nationwide survey of clinical experts in Germany
Source: Sci Rep. 2025 Jan 7;15:1043. doi: 10.1038/s41598-024-83737-1 (PMC11704235; doi:10.1038/s41598-024-83737-1)
Supplement: Supplementary file 1 — Supplementary Information 1. [file 41598_2024_83737_MOESM1_ESM.pdf]

# References for meningioma MRI reporting items

| Reporting items | Selected references |
|---|---|
| Meningioma location (epicenter) | 1 |
| Meningioma growth pattern (spherical/lobular/en plaque) | 2 |
| Meningioma extension (compartments) | 1,2 |
| Signal intensity of tumor tissue on T1WI (compared to grey matter) | 3–5 |
| Signal intensity of tumor tissue on T2WI (compared to grey matter) | 6,7 |
| Tumor contrast enhancement (intensity, homogeneity) | 1,2,8 |
| Meningioma diffusion characteristics (apparent diffusion coefficient) | 2,5,9,10 |
| Meningioma perfusion characteristics (relative cerebral blood volume) | 1,9,11,12 |
| Proton MR spectroscopic tumor profile | 8,13 |
| Nidus/Intratumoral macrovascular architecture (MR angiography) | 1,2,5 |
| Tumor calcifications (T2*/susceptibility weighted imaging) | 2,5,6,14 |
| Intratumoral necrosis | 2,15 |
| Tumor hemorrhage | 2,5 |
| Meningioma associated cysts | 2,9,14 |
| Contrast enhancement of cyst wall | 2,9 |
| Meningioma-brain interface (circumscript/invasive) | 8,16 |
| Tumor location in relation to dura mater | 17 |
| Infiltration/occlusion/thrombosis of dural venous sinuses | 8,14 |
| Perifocal edema | 1,14,15,18 |
| Adjacent bony alterations (hyperostosis/osteolysis) | 2,9 |
| Contact/encasement/constriction of arteries | 2,19 |
| Contact/encasement/displacement of cranial nerves | 19 |
| Tumor-associated hydrocephalus | 20 |
| Signs of mass effect (midline shift/ventricle compression/herniation) | 1,21 |
| Extent of resection (postoperative MRI) | 21,22 |
| Treatment-related changes (resection cavity/gliosis/postradiogenic leukoencephalopathy) | 1 |
| Nauta classification (meningioma cysts) | 23 |
| Zee classification (meningioma cysts) | 24 |
| Sindou classification (infiltration of superior sagittal sinus) | 25 |
| Simpson classification (extent of resection) | 26 |
| RANO criteria (treatment response) | 27 |

## References


1. Thust, S. & Kumar, A. Extra-axial Tumors. in *Clinical Neuroradiology* (eds. Barkhof, F., Jager, R., Thurnher, M. & Rovira Cañellas, A.) 1–37 (Springer International Publishing, Cham, 2019). doi:10.1007/978-3-319-61423-6_58-1.
2. Watts, J. *et al.* Magnetic resonance imaging of meningiomas: a pictorial review. *Insights Imaging* **5**, 113–122 (2014).
3. Chen, C.-J., Tseng, Y.-C., Hsu, H.-L. & Jung, S.-M. Microcystic Meningioma: Importance of Obvious Hypointensity on T1-Weighted Magnetic Resonance Images. *Journal of Computer Assisted Tomography* **32**, 130–134 (2008).
4. Lapolla, P. *et al.* Lipomatous Meningioma: Clinical-Pathological Findings, Imaging Characterisation and Correlations of a Rare Type of Meningioma. *In Vivo* **35**, 3031–3037 (2021).
5. Krishnan, V., Mittal, M. & Sinha, M. Imaging spectrum of meningiomas: a review of uncommon imaging appearances and their histopathological and prognostic significance. *pjr* **84**, 630–653 (2019).
6. Zeng, L., Liang, P., Jiao, J., Chen, J. & Lei, T. Will an Asymptomatic Meningioma Grow or Not Grow? A Meta-analysis. *J Neurol Surg A Cent Eur Neurosurg* **76**, 341–347 (2015).
7. Yao, A., Pain, M., Balchandani, P. & Shrivastava, R. K. Can MRI predict meningioma consistency?: a correlation with tumor pathology and systematic review. *Neurosurg Rev* **41**, 745–753 (2018).
8. Osborn, A. G. *Essentials of Osborn's Brain: A Fundamental Guide for Residents and Fellows*. (Elsevier, Philadelphia, PA, 2020).
9. Zakhari, N., Torres, C., Castillo, M. & Nguyen, T. B. Uncommon Cranial Meningioma: Key Imaging Features on Conventional and Advanced Imaging. *Clin Neuroradiol* **27**, 135–144 (2017).
10. Xiaoai, K., Qing, Z., Lei, H. & Junlin, Z. Differentiating microcystic meningioma from atypical meningioma using diffusion-weighted imaging. *Neuroradiology* **62**, 601–607 (2020).
11. Zhang, H., Rödiger, L. A., Shen, T., Miao, J. & Oudkerk, M. Perfusion MR imaging for differentiation of benign and malignant meningiomas. *Neuroradiology* **50**, 525–530 (2008).
12. Zikou, A. *et al.* The role of diffusion tensor imaging and dynamic susceptibility perfusion MRI in the evaluation of meningioma grade and subtype. *Clinical Neurology and Neurosurgery* **146**, 109–115 (2016).
13. Lin, M.-C. *et al.* Preoperative grading of intracranial meningioma by magnetic resonance spectroscopy (1H-MRS). *PLoS ONE* **13**, e0207612 (2018).
14. Alruwaili, A. A. & De Jesus, O. Meningioma. in *StatPearls* (StatPearls Publishing, Treasure Island (FL), 2023).
15. Kunimatsu, A. *et al.* Variants of meningiomas: a review of imaging findings and clinical features. *Jpn J Radiol* **34**, 459–469 (2016).
16. Upreti, T., Dube, S., Pareek, V., Sinha, N. & Shankar, J. Meningioma grading via diagnostic imaging: A systematic review and meta-analysis. *Neuroradiology* (2024) doi:10.1007/s00234-024-03404-0.
17. Nadgir, R. & Yousem, D. M. *Neuroradiology: The Requisites*. (Elsevier, Amsterdam, 2017).
18. Spille, D. C., Sporns, P. B., Heß, K., Stummer, W. & Brokinkel, B. Prediction of High-Grade Histology and Recurrence in Meningiomas Using Routine Preoperative Magnetic

Resonance Imaging: A Systematic Review. *World Neurosurgery* **128**, 174–181 (2019).
19. Adachi, K., Kawase, T., Yoshida, K., Yazaki, T. & Onozuka, S. ABC Surgical Risk Scale for skull base meningioma: a new scoring system for predicting the extent of tumor removal and neurological outcome: Clinical article. *JNS* **111**, 1053–1061 (2009).
20. Burkhardt, J.-K. *et al.* Predicting postoperative hydrocephalus in 227 patients with skull base meningioma. *FOC* **30**, E9 (2011).
21. Goldbrunner, R. *et al.* EANO guideline on the diagnosis and management of meningiomas. *Neuro-Oncology* **23**, 1821–1834 (2021).
22. Schwartz, T. H. & McDermott, M. W. The Simpson grade: abandon the scale but preserve the message. *Journal of Neurosurgery* 1–8 (2020) doi:10.3171/2020.6.JNS201904.
23. Nauta, H. J., Tucker, W. S., Horsey, W. J., Bilbao, J. M. & Gonsalves, C. Xanthochromic cysts associated with meningioma. *Journal of Neurology, Neurosurgery & Psychiatry* **42**, 529–535 (1979).
24. Zee, C. S. *et al.* Magnetic Resonance Imaging of Cystic Meningiomas and Its Surgical Implications: *Neurosurgery* **36**, 482–488 (1995).
25. Sindou, M. P. & Alvernia, J. E. Results of attempted radical tumor removal and venous repair in 100 consecutive meningiomas involving the major dural sinuses. *JNS* **105**, 514–525 (2006).
26. Simpson, D. THE RECURRENCE OF INTRACRANIAL MENINGIOMAS AFTER SURGICAL TREATMENT. *Journal of Neurology, Neurosurgery & Psychiatry* **20**, 22–39 (1957).
27. Huang, R. Y. *et al.* Proposed response assessment and endpoints for meningioma clinical trials: report from the Response Assessment in Neuro-Oncology Working Group. *Neuro-Oncology* **21**, 26–36 (2019).
