# Supplementary material for: Identification of key elements in MRI reporting of intracranial meningiomas based on a nationwide survey of clinical experts in Germany
Source: Sci Rep. 2025 Jan 7;15:1043. doi: 10.1038/s41598-024-83737-1 (PMC11704235; doi:10.1038/s41598-024-83737-1)
Supplement: Supplementary file 2 — Supplementary Information 2. [file 41598_2024_83737_MOESM2_ESM.pdf]

Structured MRI reporting template for intracranial meningiomas with a focus on the essential information needs of neuro-oncology clinicians

Spatial tumor description

- Location (e.g., skull base or convexity, intraventricular)
- Growth pattern (e.g., globose or en plaque)
- Extent (preferably three-dimensional: length x width x height)

Structural tumor characteristics

- T2 weighted imaging signal intensity
- Contrast enhancement intensity and pattern
- Intratumoral vascular architecture (e.g., marked flow voids)
- Necrotic parts?
- Hemorrhagic components?
- Tumor calcifications?
- Associated cysts (+/- enhancing wall)?

Meningioma-related complications

- Involvement/impairment of adjacent anatomical structures (e.g., brain invasion/edema, bone destruction/hyperostosis, dural venous sinus invasion, encasement/constriction of arteries or cranial nerves)?
- Mass effect (herniation)?
- Occlusive hydrocephalus?

Only for treated meningiomas

- *Following surgery:* Extent of resection (e.g., using the Meningioma Group Amsterdam (MEGA) Grading System of Meningioma Removal derived from the Simpson Grading, size of residual tumor)
- *Following any treatment:* RANO response criteria for meningiomas
- *Following radiotherapy:* Radiation-induced changes (e.g., leukoencephalopathy)?
